# Incorporating RNA-based Risk Scores for Genomic Instability to Predict Breast Cancer Recurrence and Immunogenicity in a Diverse Population

**DOI:** 10.1158/2767-9764.CRC-22-0267

**Published:** 2023-01-05

**Authors:** Alina M. Hamilton, Sarah C. Van Alsten, Xiaohua Gao, Joseph Nsonwu-Farley, Benjamin C. Calhoun, Michael I. Love, Melissa A. Troester, Katherine A. Hoadley

**Affiliations:** 1 Department of Pathology and Laboratory Medicine, School of Medicine, University of North Carolina at Chapel Hill, Chapel Hill, North Carolina.; 2 Department of Epidemiology, Gillings School of Public Health, University of North Carolina at Chapel Hill, Chapel Hill, North Carolina.; 3 Lineberger Comprehensive Cancer Center, University of North Carolina, Chapel Hill, North Carolina.; 4 Department of Biostatistics, University of North Carolina at Chapel Hill, Chapel Hill, North Carolina.; 5 Department of Genetics, Lineberger Comprehensive Cancer Center, University of North Carolina at Chapel Hill, Chapel Hill, North Carolina.

## Abstract

Markers of genomic instability, including TP53 status and homologous recombination deficiency (HRD), are candidate biomarkers of immunogenicity and immune-mediated survival, but little is known about the distribution of these markers in large, population-based cohorts of racially diverse patients with breast cancer. In prior clinical trials, DNA-based approaches have been emphasized, but recent data suggest that RNA-based assessment can capture pathway differences conveniently and may be streamlined with other RNA-based genomic risk scores. Thus, we used RNA expression to study genomic instability (HRD and TP53 pathways) in context of the breast cancer immune microenvironment in three datasets (total *n* = 4,892), including 1,942 samples from the Carolina Breast Cancer Study, a population-based study that oversampled Black (*n* = 1,026) and younger women (*n* = 1,032). Across all studies, 36.9% of estrogen receptor (ER)-positive and 92.6% of ER-negative breast cancer had presence of at least one genomic instability signature. TP53 and HRD status were significantly associated with immune expression in both ER-positive and ER-negative breast cancer. RNA-based genomic instability signatures were associated with higher PD-L1, CD8 T-cell marker, and global and multimarker immune cell expression. Among tumors with genomic instability signatures, adaptive immune response was associated with improved recurrence-free survival regardless of ER status, highlighting genomic instability as a candidate marker for predicting immunotherapy response. Leveraging a convenient, integrated RNA-based approach, this analysis shows that genomic instability interacts with immune response, an important target in breast cancer overall and in Black women who experience higher frequency of TP53 and HR deficiency.

**Significance:**

Despite promising advances in breast cancer immunotherapy, predictive biomarkers that are valid across diverse populations and breast cancer subtypes are needed. Genomic instability signatures can be coordinated with other RNA-based scores to define immunogenic breast cancers and may have value in stratifying immunotherapy trial participants.

## Introduction

Immune checkpoint blockade is rapidly changing the direction of cancer care, including breast cancer. Lung cancer and melanoma exhibit elevated immune infiltration (>50% lymphocytic infiltrate), high tumor mutation burden (≥10 mutations/Mb), and strong response to immune checkpoint blockade. In these tumor types, either high microsatellite instability, or high PD-L1 expression are markers of response for clinical use of immunotherapy (1–6). However, breast cancers rarely exhibit microsatellite instability and PD-L1 expression has been inconsistently observed in immunogenic breast tumors [i.e., only 40% of triple-negative breast cancers (TNBC) are PD-L1 positive] (7–10). Nonetheless, TNBCs are associated with relatively greater mutation load (8, 11) and high tumor-infiltrating lymphocytes (TIL; refs. 12, 13). As such, TNBC status has been proposed as a candidate biomarker indicating benefit from immune checkpoint inhibitor pembrolizumab; pembrolizumab has been approved for PD-L1–positive metastatic TNBC (14) and early-stage II/III TNBC (15). Unfortunately, an exclusive focus on TNBC may miss immunogenic estrogen receptor (ER)-positive breast cancers, a particular concern among Black patients, who have a higher frequency of basal-like breast cancer among ER-borderline (16) and who have higher rates of some types of genomic instability (17, 18).

We hypothesized that breast-specific indicators of genomic instability may be combined with other RNA-based risk markers to identify candidate markers predictive of immune response. Given the prevailing theory that genomic instability produces neoantigens to elicit an adaptive anticancer immune response (1, 6, 19, 20), two key pathways are of interest: *TP53* is the most frequently occurring mutation in breast cancer (11) and homologous recombination deficiency (HRD) occurs at increased frequencies in breast cancer, second only to ovarian cancer in a pan-cancer comparison (21, 22). Both pathways are associated with immune response in cancer (23–25). Furthermore, Black women and younger women have more frequent *TP53* mutations and more frequent HRD (26, 27). We have shown that RNA-based assessment of TP53 and HRD pathways can sensitively detect DNA repair differences (17, 26). Thus, we evaluated RNA-based signatures for TP53 and HRD in association with tumor immune profiles in 1,942 patients from the Carolina Breast Cancer Study (CBCS; *n* = 1,026 Black, 916 non-Black), with validation in two publicly available datasets [the Cancer Genome Atlas (TCGA; *n* = 1,046) and the Molecular Taxonomy of Breast Cancer International Consortium (METABRIC; *n* = 1,904)]. In addition, we assessed markers of genomic instability as modifiers of immune-mediated recurrence outcomes in the CBCS.

## Materials and Methods

### Study Populations

The CBCS has been described in previous publications (28). In brief, CBCS is a three-phase population-based study that utilized rapid case ascertainment in cooperation with the North Carolina Central Cancer Registry to identify women ages 20–74 years diagnosed with first primary invasive breast cancer. Phase I and II of CBCS enrolled participants from 1993 to 2001 across 24 counties, and phase III expanded the study to 44 counties between 2008 and 2013. Black and younger women (ages <50 years) were oversampled using randomized recruitment. In total, there are 4,806 invasive breast cancer cases who were enrolled in the CBCS (phases I–III). A total of 1,188 participants were removed because of inadequate tissue amounts for analysis, and an additional 241 participants were removed because of low-quality RNA. Of the remaining 3,377 participants, 1,952 cases (phase I: *n* = 252, phase II: *n* = 454, phase III: *n* = 1,246) were analyzed with a custom NanoString expression panel, and 10 samples were excluded in the current analysis due to missing ER status. Clinical and demographic features of the analysis set were similar to that of the CBCS as a whole, except higher grade (36.8% vs. 30.8%) and node-positive tumors (43.2% vs. 38.5%). This study was approved by the University of North Carolina at Chapel Hill Office of Human Ethics and Institutional Review Board.

Two publicly available datasets were used: TCGA breast cancer dataset and the METABRIC. TCGA is a worldwide study with fresh-frozen tissue collected for breast tumors from 40 sites worldwide (*N* = 1,095). In the current study, 46 TCGA tumors were excluded because of missing or indeterminate ER status, resulting in 1,046 cases for analysis. METABRIC is a collection of fresh-frozen primary breast tumors obtained from five tumor banks across the United Kingdom and Canada from 1977 to 2005 (*N* = 1,904). Tumor and patient characteristics for all three studies are described in Table 1 and Supplementary Table S1, respectively.

**TABLE 1:** Distribution of TP53 functionality, HRD status, and immune expression according to ER status across three studies

	CBCS		TCGA		METABRIC	
	ER-Negative	ER-Positive	ER-Negative	ER-Positive	ER-Negative	ER-Positive
**Total**	714	1,228	239	807	445	1,459
	*n* (%)	*n* (%)	*n* (%)	*n* (%)	*n* (%)	*n* (%)
**TP53**						
WT-like	96 (13.4)	930 (75.7)	15 (6.3)	524 (64.9)	19 (4.3)	1,024 (70.2)
Mut-like	612 (85.7)	290 (23.6)	224 (93.7)	283 (35.1)	426 (95.7)	435 (29.8)
Missing	6 (0.8)	8 (0.7)				
**HRD** ^a^						
HRD−	83 (16.5)	710 (77.6)	30 (12.6)	617 (76.5)	81 (18.2)	1023 (70.1)
HRD+	421 (83.5)	205 (22.4)	209 (87.4)	190 (23.5)	364 (81.8)	436 (29.9)
**Any genomic instability** ^a^						
NGI	50 (7.4)	626 (63.1)	13 (5.5)	509 (63.1)	19 (4.3)	919 (63.0)
AGI	628 (92.6)	495 (36.9)	224 (94.5)	298 (36.9)	426 (95.7)	540 (37.0)
**Immune phenotype**						
Immune-Quiet	85 (11.9)	431 (35.1)	4 (1.7)	66 (8.2)	94 (21.1)	390 (26.7)
Innate-Enriched	301 (42.2)	511 (41.6)	103 (43.1)	407 (50.4)	67 (15.1)	495 (33.9)
Adaptive-Enriched	328 (45.9)	286 (23.3)	132 (55.2)	334 (41.4)	284 (63.8)	574 (39.3)
**PD-L1**						
Low	562 (78.7)	1,055 (85.9)	142 (59.4)	641 (79.4)	280 (62.9)	1,148 (78.7)
High	152 (21.3)	173 (14.1)	97 (40.6)	166 (20.6)	165 (37.1)	311 (21.3)
**CD8 T cell**						
Low	493 (69)	963 (78.4)	153 (64)	632 (78.3)	261 (58.7)	1,167 (80)
High	221 (31)	265 (21.6)	86 (36)	175 (21.7)	184 (41.3)	292 (20)

Abbreviations: CBCS: Carolina Breast Cancer Study; ER: estrogen receptor; IHC: immunohistochemistry; METABRIC: Molecular Taxonomy of Breast Cancer International Consortium; NA: Not available; TCGA: the Cancer Genome Atlas.

^a^HRD classifier not available for 210 ER-negative and 313 ER-positive samples in CBCS. As a result, AGI measurements were not available for 36 ER-negative and 234 ER-positive cases.

### Demographics, Clinical Characteristics, and Tumor Markers

In CBCS, health history, demographic variables, and body measurements were collected by a nurse during in-home interviews. Race was self-reported and categorized as non-Black or Black; <5% of non-Black participants self-identified as multiracial, Hispanic, or other race/ethnicity. Genetic ancestry and self-reported race are strongly concordant in CBCS (29); however, we interpret race as a social construct, where race-specific differences represent molecular and cellular effects resulting from the culmination of common genetic variation, individual, community, and structural factors that differ by self-reported race. Tumor size, American Joint Committee on Cancer stage, ER, progesterone receptor, and HER2 receptor status were obtained from medical records, pathology reports and IHC staining performed at University of North Carolina at Chapel Hill. In METABRIC and TCGA, IHC-based hormone receptor status and other tumor variables (tumor stage and size) were obtained from the medical records.

### Recurrence Data

Recurrence data were available for CBCS phase III (2008–2013; *n*  =  1246) through patient follow-up and subsequent medical record confirmation. Time to recurrence was defined as the time between date of diagnosis to first local, regional, or distant recurrent breast and verified through medical record review. Recurrence data are complete through October 2019 with 5-year follow-up for all women. Among 1246 eligible women, 47 participants were stage IV at diagnosis and excluded from recurrence analysis. Among 1,199 patients (stage I–III), 143 recurrences were identified.

### Gene Expression Data

#### Normalization, Molecular Subtyping, Global Immune Classes, and Immune Scores

NanoString gene expression data in CBCS was collected and normalized using Remove Unwanted Variation (30) as described previously (31, 32), along with generation of PAM50 molecular subtypes, global immune classes and 10 immune cell scores. Briefly, a research version of the PAM50 molecular subtype predictor was used to classify tumors as luminal A, luminal B, HER2-enriched, basal-like or normal-like, and to generate risk of recurrence scores (ROR-PT) incorporating tumor size, proliferation, and subtype. Global immune classes were identified using a 48-gene panel of immune cell–related genes and hierarchical clustering analysis (31). We had previously identified three global immune expression classes reflecting adaptive-enriched, innate-enriched, and immune-quiet tumor microenvironments using consensus clustering (33). A classifier of CBCS immune classes was developed and applied to TCGA and METABRIC breast cancer expression datasets using Classification to the Nearest Centroid (34). CBCS global immune classes had high concordance with TCGA's pan-cancer immune subtypes (19) [see Supplementary Fig. S3 in Hamilton and colleagues, 2022 (31)].

In addition, 10 major immune cell scores (B-cell, T-cell, CD8-T cell, Th cell, regulatory T cell, T follicular helper (Tfh) cells, eosinophil, neutrophil, natural killer, and macrophage) and a cytotoxic cell, adaptive cell, innate cell and PD-L1 score, were generated by computing the median cell-type specific gene expression as described previously (31). Markers for these immune cell types were selected on the basis of work from Bindea and colleagues, 2013 (35) and Danaher and colleagues, 2017 (36), which respectively utilized whole transcriptome and targeted RNA expression methods from immune cell and bulk tumor samples to establish marker panels, and subsequently validated against flow cytometry and IHC data. PD-L1 (*CD274*) and CD8 T-cell expression (*CD8A*) were categorized as low versus high using the third quartile as a cut-off point. Within the CBCS, RNA markers were further validated against immunofluorescence (IF) and hematoxylin and eosin–based quantification of TILs on tumor slides from the same block. RNA profiling was completed using tumor cores so the correlations are per tumor and not per specimen, but reflect strong concordance (e.g., RNA adaptive-cell score vs. TILs: rho = 0.29, *P* = 2.2e-16; ref. 31). Similarly high correlations were confirmed between the B-cell score and IF for CD19 [RNA B-cell score vs. IF CD19: rho = 0.75, *P* = 5.3e-9; see Supplementary Fig. S1 from Hamilton and colleagues, 2022 (31)].

#### TP53 Functional Phenotypes and HRD Classifier

To identify tumors with genomic instability, we used published RNA-based signatures to classify tumors by TP53 and HRD status. Specifically, tumors were classified as TP53 mutant-like (Mut-like) or wildtype (WT)-like using a similarity-to-centroid approach (26, 37). HRD-high status was determined (17) using a classifier based on TCGA HRD status (which included HRD-LOH, large-scale transitions, and the number of subchromosomal regions with allelic imbalance extending to the telomere). This classifier has been published previously (17) and was applied to both CBCS and METABRIC datasets (17).

### Statistical Analysis

Associations between genomic instability classifiers and immune classes were assessed using Welch two-sample *t* tests. Generalized linear models were used to calculate relative frequency differences (RFD) and 95% confidence intervals (CI) as the measure of association. RFDs can be interpreted as an estimate of the percentage difference between index and referent groups, in this case, the relative frequency of immune classes according to categories of genomic instability. When estimating frequency differences by clinical and demographic characteristics, multivariable models were adjusted for age and race.

Kaplan–Meier curves and log-rank tests were used to compare mean time to recurrence across global immune classes in stage I–III CBCS cases (*n* = 1,199). Recurrence analyses were stratified by presence of any genomic instability (AGI), defined as tumors with either TP53 Mut-like or HRD-high classification, or no genomic instability (NGI; i.e., presence of neither phenotype). HRs and 95% CI were calculated using Cox proportional hazards models overall, and adjusted for age, race, tumor stage, and ER status. The assumption of proportionality was assessed via the Wald *P* value. Point estimates from models that included covariate–time interaction terms did not vary substantially. All statistical analyses were performed in R version 4.0.3.

### Data Availability

TCGA mRNA-sequencing expression data are publicly available at the Genome Data Commons under dbGaP accession phs000178.v1.p1. Additional data are available at https://gdc.cancer.gov/about-data/publications/PanCan-CellOfOrigin (38). All primary METABRIC data, deposited at the European Genome-phenome Archive (EGA) (EGAS00000000083), are available upon request from the METABRIC Data Access Committee. RNA microarray gene expression data from the original METABRIC publication (39) can be found on cBioPortal at https://www.cbioportal.org/study/summary?id=brca_metabric. CBCS data are available upon request (https://unclineberger.org/cbcs).

## Results

We evaluated genomic instability in association with immune classes in three datasets [CBCS (*n* = 1,942), TCGA breast cancer (*n* = 1,046), and METABRIC (*n* = 1,904)], which differed according to clinical and demographic variables. Compared with TCGA and METABRIC, CBCS had a larger proportion of younger participants (<50 years), with median patient ages of 49, 58, and 61.2 years in CBCS, TCGA, and METABRIC, respectively. In addition, the CBCS had a higher proportion of Black patients (52%) than METABRIC (0%) and TCGA (17%; Supplementary Table S1). ER-positive tumors were more prevalent in TCGA and METABRIC populations. The distribution of tumor stage also differed between the three studies, where TCGA had the highest proportion of advanced-stage tumors, particularly among ER-positive cases. Conversely, METABRIC had the lowest fraction of high-stage among both ER-positive and ER-negative tumors (Supplementary Table S1).

Across all datasets, genomic instability signatures (TP53 Mut-like or HRD-high status) were more prevalent among ER-negative tumors but occurred in a sizable proportion of ER-positive tumors. Among ER-positive cases in CBCS, 22.4% had HRD-high, 23.6% were TP53 Mut-like, and 36.9% had presence of either signature (compared with 83.5% HRD-high, 85.7% TP53 Mut-like, 92.6% with either signature among ER-negatives). Consistent with previous literature (13), adaptive-enriched immune class, PD-L1-high and CD8 T cell-high gene expression were more prevalent among ER-negative tumors than ER-positive tumors across all three studies (Table 1).

**FIGURE 1:**
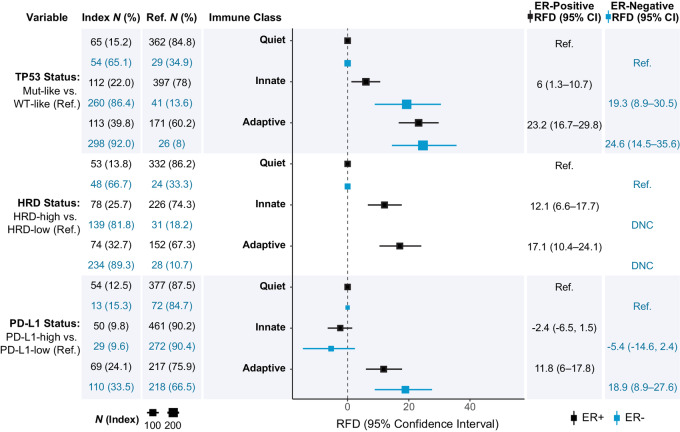
Association between CBCS immune classes, TP53, HRD, and PD-L1 status. Forest plot displaying relative frequency differences and 95% CIs for TP53, HRD, and PD-L1 status across global immune classes, adjusted for age and race. Analyses were stratified by ER status, with associations for ER-positive tumors represented by black points and ER-negative tumors represented by blue points. 95% CI: 95% confidence interval; DNC: does not converge; ER: estrogen receptor; RFD: relative frequency difference. Referent group = Immune-quiet for all models.

**FIGURE 2:**
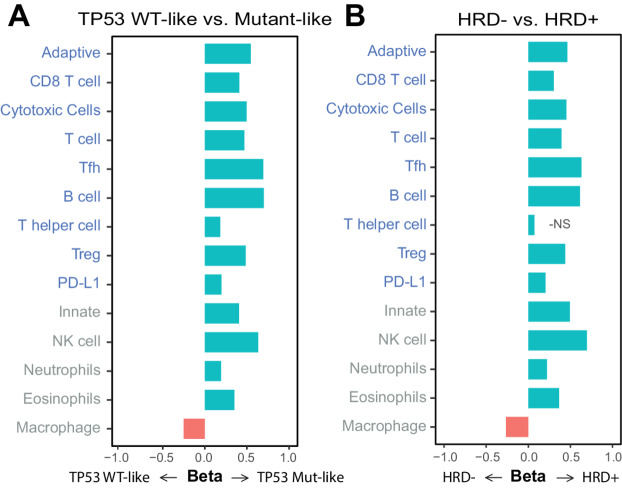
Differential immune cell score analysis by TP53 and HRD status. Horizontal bar plots displaying beta values from generalized linear models for immune cell scores for TP53 Mut-like versus WT-like tumors (referent: WT-like; **A**) and HRD-high versus HRD-low tumors (referent: HRD-low; **B**). The text for adaptive cell scores are labeled in blue and innate cell scores are labeled in gray. All analyses were adjusted for patient age and race. *P* values were adjusted for multiple testing using the Benjamani–Hochburg procedure. NS: not statistically significant.

In an ER-stratified analyses adjusted for age and race, we evaluated associations between TP53 Mut-like, HRD-high, PD-L1-high status (independent variables), and previously defined immune classes and immune cell scores (dependent variable). Adaptive-enriched immune class was associated with TP53 Mut-like, HRD-high, and PD-L1-high and innate-enriched class was associated with TP53 Mut-like and HRD-high only (Fig. 1). Associations were significant overall and in strata defined by ER-positive status, except for HRD among ER-negatives, which failed to converge due to sparse numbers of low-HRD, immune-quiet tumors. Differential expression of immune cell scores by TP53 or HRD status identified higher expression of both adaptive and innate cell types in TP53 Mut-like and HRD-high except for Macrophages in both, and a nonsignificant association of T helper cells in HRD (Fig. 2A and B). Associations for TP53 status remained significant regardless of ER status but were limited to ER-positive cases for HRD (Supplementary Fig. S1), in part due to low proportions of HRD-low, ER-negative tumors (16.5%; Table 1). Associations between adaptive and innate immune scores, TP53 status, HRD status, and PD-L1 class can be seen in Supplementary Fig. S2, and beta values and *P* values for immune associations are presented in Supplementary Table S2.

**FIGURE 3:**
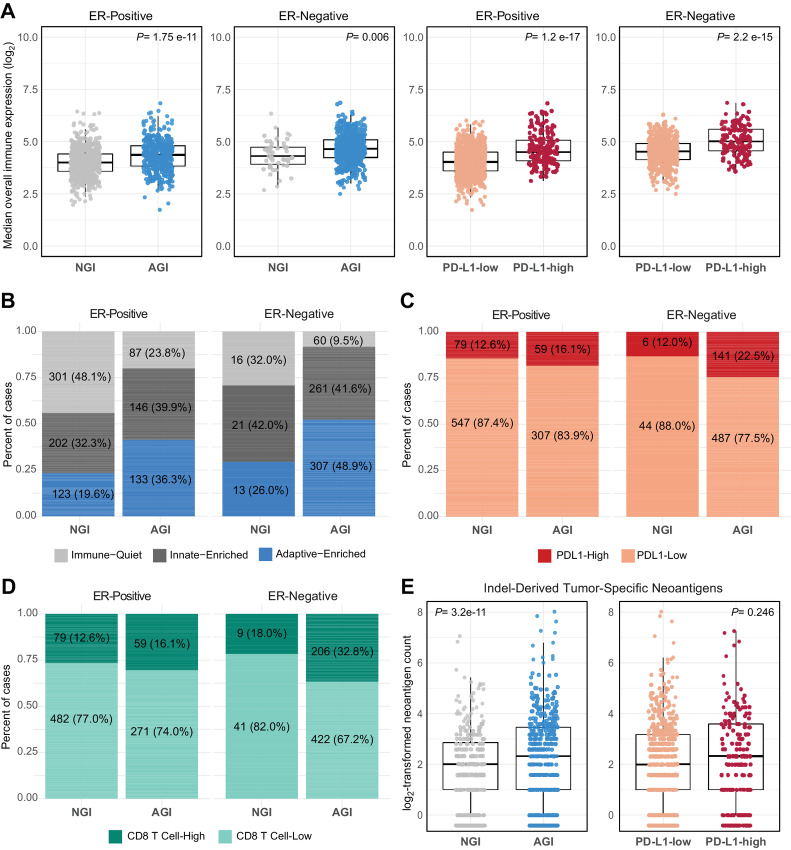
Distribution of immune classes, PD-L1 and CD8 T-cell categories by AGI and ER status. **A,** Boxplot displaying overall immune expression levels, calculated as the median of all 48 immune genes, grouped by AGI versus NGI tumors, and PD-L1-high versus PD-L1-low, stratified by ER status (ER-positive: left; ER-negative: right). Stacked bar plots displaying PDL1-high (red) or PDL1-low (orange; **C**), the proportion of tumors classified as immune-quiet (light gray), innate-enriched (dark gray) or adaptive-enriched (blue; **B**), and CD8 T cell-high (dark green) or CD8 T cell-low (light green) among AGI and NGI tumors (**D**), stratified by ER status (ER-positive: left; ER-negative: right). Number and percent of samples [*n* (%)] are displayed within bars (**A–D**). **E,** Boxplot displaying number of indel-derived neoantigens in AGI versus NGI tumors (left), and PD-L1-high versus PD-L1-low tumors (right). CBCS data shown in **A**–**D**; TCGA data are shown in **E**.

We investigated whether any genomic instability (AGI), defined as either TP53 Mut-like or HRD-high, was associated with immune response and PD-L1 status. Among both ER-positive and ER-negative cases in CBCS, AGI tumors had significantly elevated overall immune expression, similar to PD-L1-high tumors (Fig. 3A), as well as higher proportions of adaptive-enriched class (Fig. 3B), higher proportions of PD-L1-high tumors (Fig. 3C), and higher proportions of CD8 T cell-high tumors (Fig. 3D). Associations observed in CBCS between AGI and immune classes were replicated among TCGA and METABRIC cases, with higher proportions of adaptive-enriched, PD-L1-high, and CD8 T cell-high among AGI tumors, regardless of ER status (Supplementary Fig. S3).

As neoantigens are implicated in antitumor immune response, we used previously quantified insertion/deletion (indel)-derived neoantigens (19) to investigate the relationship between pathway-based AGI and neoantigen levels in TCGA. AGI tumors had significantly higher neoantigen count (Fig. 3E; left), but no difference in neoantigen load was observed between PD-L1-high and PD-L1-low (Fig. 3E; right). Collectively, these data show that AGI and PD-L1 both associate with elevated immune expression, but approximately 80% of AGI cases were PD-L1-low, while approximately 12% of cases with no genomic instability (NGI) were identified as PD-L1-high (Fig. 3C), highlighting the importance of both markers for capturing immunogenic breast cancers.

**FIGURE 4:**
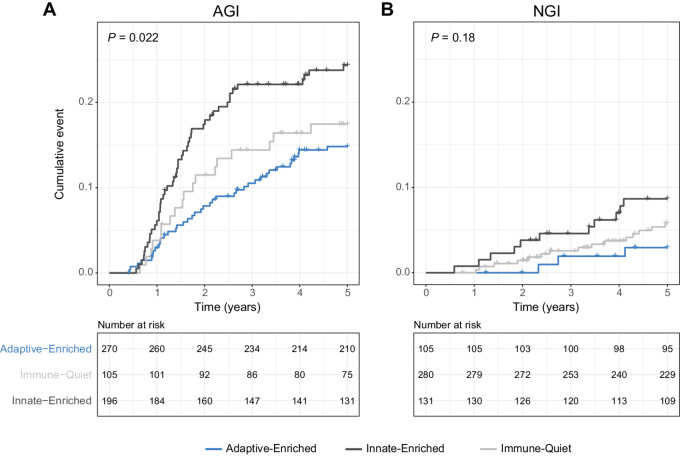
Immune-mediated survival by global immune classes in CBCS. Kaplan–Meier survival analysis illustrating 5-year RFS among CBCS phase III AGI cases (**A**) and NGI cases (**B**). All analyses were restricted to stage I–III tumors. Tick marks represent censored individuals. Log-rank *P* value in top left of each plot.

Finally, we examined the association between global immune classes and recurrence-free survival (RFS) according to AGI in CBCS. Previously (31), we found that relationships between global immune classes and recurrence were limited to ER-negative tumors, with the best RFS observed among tumors with an adaptive-enriched microenvironment, and the poorest RFS observed among innate-enriched tumors [innate-enriched HR (95% CI): 1.78 (1.09–2.91); Supplementary Table S3]. However, in the current analysis, we used AGI to stratify our data. Significant differences were observed by global immune classes among AGI tumors, with the best RFS observed among adaptive-enriched tumors, and poorest RFS among innate-enriched tumors. There was no significant difference in RFS between immune classes among NGI tumors (Fig. 4). Multivariate Cox proportional hazards models of AGI tumors remained significant after adjusting for age, race, tumor stage, and ER status [innate-enriched HR (95% CI): 1.79 (1.17–2.75); Table 2].

**TABLE 2:** Risk of recurrence for global immune classes among breast tumors with AGI

	**CBCS AGI cases (*N* = 704)**		
Immune phenotype	No. of events/N	Crude HR (95% CI)	HR^a^ (95% CI)
Adaptive-Enriched	40/328	1.00	1.00
Innate-Enriched	52/219	1.94 (1.27–2.97)	1.79 (1.17–2.75)
Immune-Quiet	28/157	1.20 (0.71–1.27)	1.65 (0.97–2.83)

Abbreviations: 95% CI: 95% confidence interval; CBCS: Carolina Breast Cancer Study; HR: hazard ratio.

^a^Adjusted for age, race, stage, and estrogen receptor status.

## Discussion

In this analysis, markers of genomic instability were associated with tumor immune microenvironments across three large studies representing a total of 4,892 patients with breast cancer, including a large study population of Black and younger patients from the CBCS. The latter population is important given higher proportions of TP53 (26) and HRD (17) signatures in younger women and Black women. TP53 and HRD status were each associated with robust immune response in breast cancer and having either signature (vs. neither) modified the association between immune status and recurrence. These findings suggest that RNA-based signatures may be candidate markers for immunotherapy response, including the capability to detect a large proportion of immunogenic tumors that are not triple negative. Given the higher proportion of basal-like breast cancer among clinically ER^+^/HER2^−^ and ER-borderline tumors in Black women (16), RNA-based measures for predicting immunotherapy could be particularly clinically meaningful among diverse populations.

Our previous work showed that immune classes were prognostic among ER-negative, but not ER-positive patients (31). The current work extended the population within which prognostication was significant to include additional ER-positive cases who had RNA-based evidence of genomic instability (40% of ER-positives; *N* = 228/571). If validated, this could extend eligible patients substantially. This marker could also be used in combination with other markers. For example, previous studies have shown that PD-L1 status and tumor mutation burden independently predict response to immune checkpoint blockade (40) and therefore may have additive value. Given limitations in tumor mutation burden as a biomarker (41), especially in breast cancer, a pathway-based approach has appealing advantages. In our data, both PD-L1-high and AGI were associated with adaptive immune response, and increased neoantigens from immunogenic indels were associated with AGI tumors, with no corresponding difference in neoantigen levels between PD-L1-high and PD-L1-low tumors.

Our results should be interpreted in context of previous studies. A previous melanoma study identified a negative association between TP53-associated aneuploidy and response to CTLA-4 inhibitors (42), while studies in non–small cell lung cancer and esophagogastric adenocarcinoma tissue have found *TP53* and HR mutations predicted response to immune checkpoint blockade (43, 44). These differences across cancer sites underscore that predictive markers may vary by tissue type. Similar to our previous work, others have identified a relationship between immune microenvironments and patient outcomes focused only on ER-negative breast cancers (12, 13, 31, 45, 46). These studies have not evaluated AGI, but promising data from the ISPY-2 trial recently identified a set of ER-positive tumors that responded to immune checkpoint blockade (47). This suggests that some ER-positive cases benefit from immunotherapy. Exploration of these biomarkers in retrospective studies of breast cancer immune checkpoint blockade and in diverse populations is an important future direction.

A strength of our analysis was the use of three breast cancer datasets, including a large, population-based cohort enriched for younger women and Black patients, a broad lens for immunogenicity based on 48 markers, and use of validated signatures for genomic instability. However, there are also some limitations of our work. Our data were drawn from observational studies, not clinical trials. As such, participants were heterogeneously treated. At present, few breast cancer immunotherapy trials have accessible data for evaluating novel predictive biomarkers, and thus correlative trials are an important first step. Given that both HRD and *TP53* mutations are associated with race in both ER-negative and ER-positive breast cancer (26, 27), and strong immunogenicity in breast tumors from Black patients (31), novel predictive markers are an unmet clinical need. While DNA-based measures offer high specificity in the detection of *TP53* and HR-related mutations, RNA-based assessment provides a more sensitive method for detecting meaningful functional differences in the context of immune response, while offering additional options for formalin-fixed paraffin-embedded material. RNA-based profiling is now widely used for prognostication and for chemotherapy decision making, and thus, an expanded set of RNA-based targets may represent an efficient way to track joint immunomodulatory and genomic instability pathways for improved prediction of immunotherapy response.

## Supplementary Material

Supplemental Figure SF1 Supplemental Figure 1 shows differential expression analysis of immune scores according to TP53 and HRD status, stratified by estrogen receptor status in CBCS.

Supplemental Figure SF2 Supplemental figure 2 displays unadjusted adaptive and innate scores according TP53, HRD and PD-L1 status, stratified by estrogen receptor status.

Supplemental Figure SF3 Supplemental figure 3 shows the distribution of immune classes, PD-L1 and CD8 T-cell categories by AGI status in ER-positive and ER-negative tumors in TCGA and METABRIC.

Supplemental Table ST1 Supplemental table 1 displays tumor and patient characteristics by estrogen receptor status in CBCS, TCGA and METABRIC.

Supplementary Table ST2 Beta values and p-values for immune associations

Supplemental Table ST3 Supplemental table 3 shows five-year recurrence hazard ratios for immune classes among ER-negative and ER-positive cases in CBCS.
