# Supplementary material for: Incorporating RNA-based Risk Scores for Genomic Instability to Predict Breast Cancer Recurrence and Immunogenicity in a Diverse Population
Source: Cancer Res Commun. 2023 Jan 5;3(1):12–20. doi: 10.1158/2767-9764.CRC-22-0267 (PMC10035450; doi:10.1158/2767-9764.CRC-22-0267)
Supplement: Supplemental Figure SF1 — Supplemental Figure 1 shows differential expression analysis of immune scores according to TP53 and HRD status, stratified by estrogen receptor status in CBCS. [file crc-22-0267-s01.docx]

**
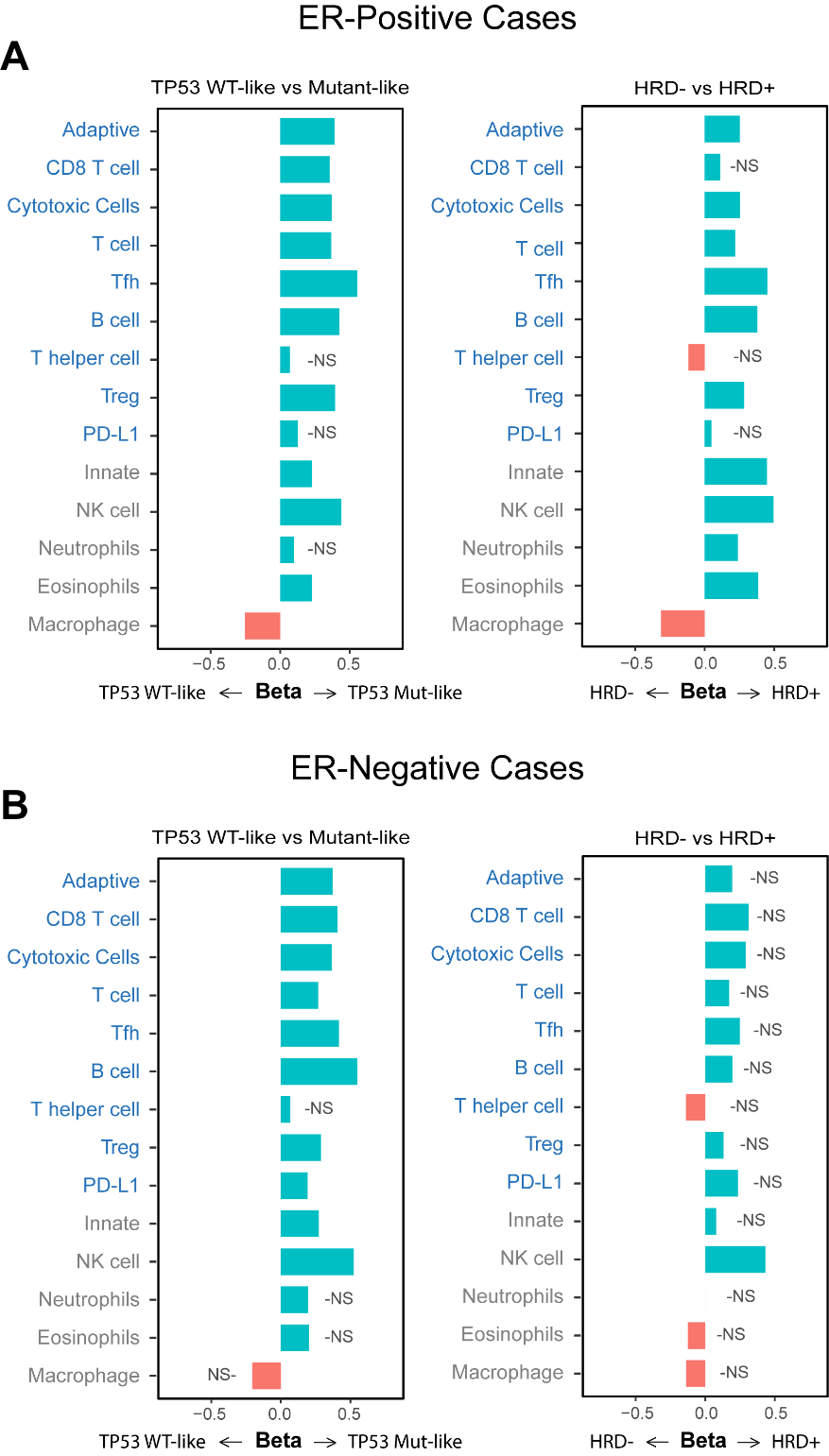
Supplemental Figure 1. Differential immune cell score analysis by TP53 and HRD status, stratified by estrogen receptor status.** Horizontal bar plots displaying beta values from generalized linear models for immune cell scores for TP53-mut like vs WT-like tumors (referent: WT-like; left panel), HRD-high vs HRD-low tumors (referent: HRD-low; right panel) among **A)** ER-positive tumors **B**) ER-negative tumors. All analyses were adjusted for patient age and race. P-values were adjusted for multiple testing using the Benjamani-Hochburg procedure. Adaptive cell scores are labeled in blue, while innate scores were labeled in grey. ER: Estrogen Receptor; NS: Not statistically significant.
