# Supplementary material for: Incorporating RNA-based Risk Scores for Genomic Instability to Predict Breast Cancer Recurrence and Immunogenicity in a Diverse Population
Source: Cancer Res Commun. 2023 Jan 5;3(1):12–20. doi: 10.1158/2767-9764.CRC-22-0267 (PMC10035450; doi:10.1158/2767-9764.CRC-22-0267)
Supplement: Supplemental Figure SF2 — Supplemental figure 2 displays unadjusted adaptive and innate scores according TP53, HRD and PD-L1 status, stratified by estrogen receptor status. [file crc-22-0267-s02.docx]

**
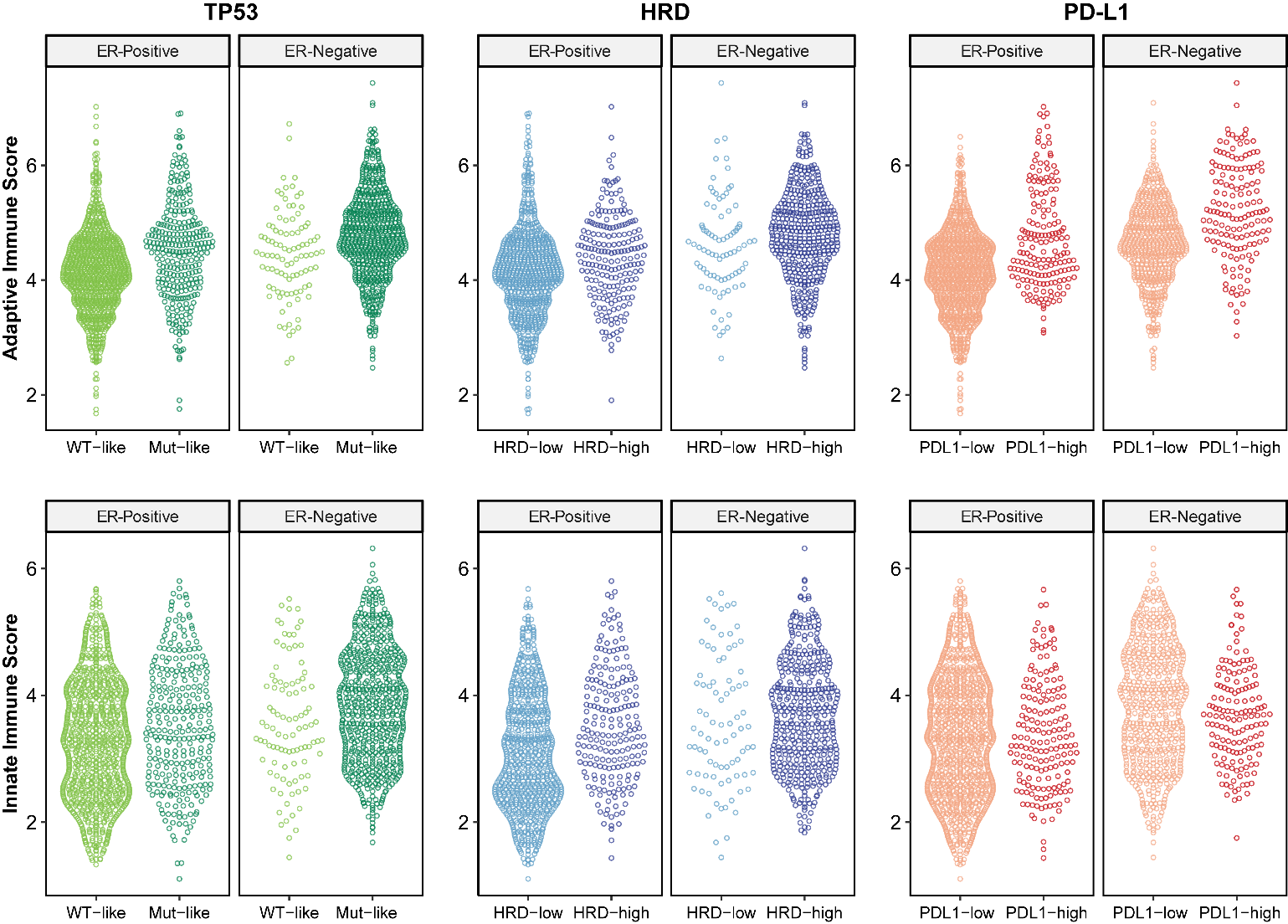
**

**Supplemental Figure 2. Unadjusted adaptive and innate immune score distributions across TP53, HRD, and PD-L1 classes, stratified by estrogen receptor status.** Bee swarm plots displaying the distribution of unadjusted adaptive immune scores (upper row) and innate immune scores (lower row) according to TP53 functional status (left panel), HRD status (middle panel) and PD-L1 class (right panel), among ER-positive and ER-negative breast tumors (ER-positive: left; ER-negative: right). ER: Estrogen Receptor; HRD: Homologous Recombination Deficiency.
