# Supplementary material for: Incorporating RNA-based Risk Scores for Genomic Instability to Predict Breast Cancer Recurrence and Immunogenicity in a Diverse Population
Source: Cancer Res Commun. 2023 Jan 5;3(1):12–20. doi: 10.1158/2767-9764.CRC-22-0267 (PMC10035450; doi:10.1158/2767-9764.CRC-22-0267)
Supplement: Supplemental Figure SF3 — Supplemental figure 3 shows the distribution of immune classes, PD-L1 and CD8 T-cell categories by AGI status in ER-positive and ER-negative tumors in TCGA and METABRIC. [file crc-22-0267-s03.docx]

**
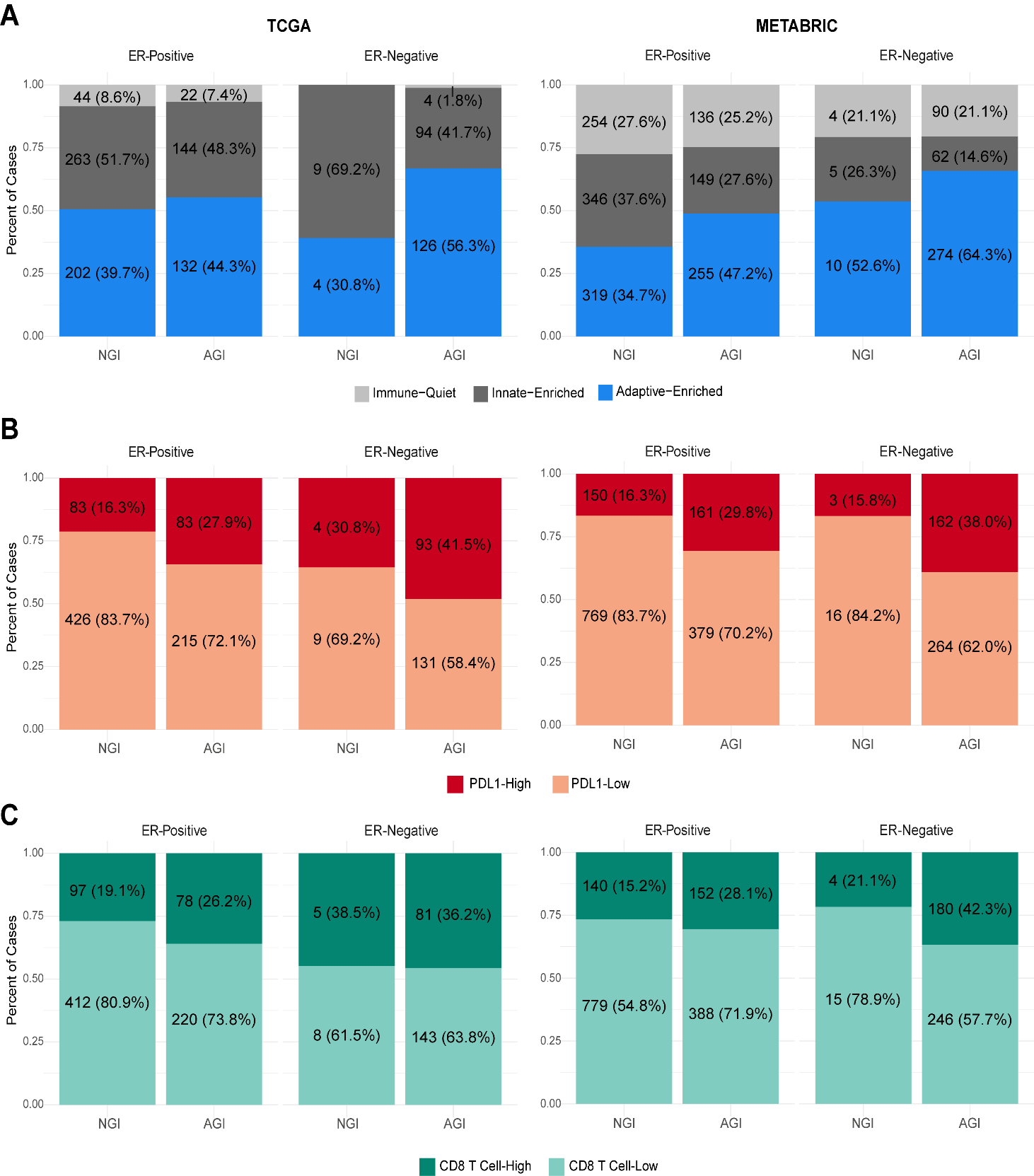
Supplemental Figure 3. Distribution of Immune Phenotypes, PD-L1 and CD8 T-Cell categories by AGI and estrogen receptor status in TCGA and METABRIC.** Stacked bar plots displaying **A**) the proportion of tumors classified as immune-quiet (light grey), innate-enriched (dark grey) or adaptive-enriched (blue), **B**) PDL1-high (red) or PDL1-low (orange), and **C**) CD8 T cell-high (dark green) or CD8 T cell-low (light green) among AGI and NGI tumors, stratified by ER status (ER-positive: left; ER-negative: right) among TCGA and METABRIC tumors. Number and percent of samples [n (%)] are displayed within bars. ER: estrogen receptor; AGI: any genomic instability; NGI: no genomic instability.
