# Supplementary material for: Incorporating RNA-based Risk Scores for Genomic Instability to Predict Breast Cancer Recurrence and Immunogenicity in a Diverse Population
Source: Cancer Res Commun. 2023 Jan 5;3(1):12–20. doi: 10.1158/2767-9764.CRC-22-0267 (PMC10035450; doi:10.1158/2767-9764.CRC-22-0267)
Supplement: Supplemental Table ST1 — Supplemental table 1 displays tumor and patient characteristics by estrogen receptor status in CBCS, TCGA and METABRIC. [file crc-22-0267-s04.docx]

| **Supplemental Table 1. Tumor and patient characteristics by estrogen receptor status across three studies** | | | | | | | | | | | | |
| --- | --- | --- | --- | --- | --- | --- | --- | --- | --- | --- | --- | --- |
| ­ |  | **CBCS** | |  | **TCGA** | | |  | | **METABRIC** | | |
|  |  | **ER-Negative** | **ER-Positive** |  | | **ER-Negative** | **ER-Positive** | |  | | **ER-Negative** | **ER-Positive** |
| **Total** |  | 714 | 1228 |  | | 239 | 807 | |  | | 445 | 1459 |
|  |  | n (%) | n (%) |  | | n (%) | n (%) | |  | | n (%) | n­ (%) |
| **Age** |  |  |  |  | |  |  | |  | |  |  |
| <50 years |  | 435 (60.9) | 597 (48.6) |  | | 76 (31.8) | 201 (24.9) | |  | | 170 (38.2) | 241 (16.5) |
| ≥50 years |  | 279 (39.1) | 631 (51.4) |  | | 163 (68.2) | 605 (75) | |  | | 275 (61.8) | 1218 (83.5) |
| Missing |  |  |  |  | | - | 1 (0.1) | |  | |  |  |
| **Race** |  |  |  |  | |  |  | |  | |  |  |
| Black |  | 471 (66) | 555 (45.2) |  | | 69 (28.9) | 110 (13.6) | |  | | NA | NA |
| non-Black |  | 243 (34) | 673 (54.8) |  | | 159 (66.5) | 613 (76) | |  | | NA | NA |
| Missing |  |  |  |  | | 11 (4.6) | 84 (10.4) | |  | |  |  |
| **Stage** |  |  |  |  | |  |  | |  | |  |  |
| Stage I/II |  | 564 (79) | 1041 (84.8) |  | | 183 (76.6) | 583 (72.2) | |  | | 273 (85.3) | 1002 (92.9) |
| Stage III/IV |  | 145 (20.3) | 176 (14.3) |  | | 50 (20.9) | 207 (25.7) | |  | | 47 (14.7) | 77 (7.1) |
| Missing |  | 5 (0.7) | 11 (0.9) |  | | 6 (2.5) | 17 (2.1) | |  | | 125* | 380* |
| **PR IHC** |  |  |  |  | |  |  | |  | |  |  |
| Positive |  | 62 (8.7) | 964 (78.5) |  | | 17 (7.1) | 681 (84.4) | |  | | 19 (4.3) | 990 (67.9) |
| Negative |  | 649 (90.9) | 259 (21.1) |  | | 221 (92.5) | 126 (15.6) | |  | | 426 (95.7) | 469 (32.1) |
| Missing |  | 3 (0.4) | 5 (0.4) |  | | 1 (0.4) | - | |  | |  |  |
| **HER2 IHC** |  |  |  |  | |  |  | |  | |  |  |
| Positive |  | 128 (17.9) | 172 (14) |  | | 41 (17.2) | 123 (15.2) | |  | | 132 (29.7) | 104 (7.1) |
| Negative |  | 580 (81.2) | 1047 (85.3) |  | | 163 (68.2) | 589 (73) | |  | | 313 (70.3) | 1355 (92.9) |
| Missing |  | 6 (0.8) | 9 (0.7) |  | | 35 (14.6) | 95 (11.8) | |  | |  |  |
| **PAM50** |  |  |  |  | |  |  | |  | |  |  |
| Luminal A |  | 63 (8.8) | 783 (63.8) |  | | 11 (4.6) | 532 (65.9) | |  | | - | 517 (35.4) |
| Luminal B |  | 27 (3.8) | 280 (22.8) |  | | 3 (1.3) | 202 (25) | |  | | - | 453 (31) |
| HER2-E |  | 114 (16) | 63 (5.1) |  | | 49 (20.5) | 29 (3.6) | |  | | 122 (27.4) | 146 (10) |
| Basal-like |  | 474 (66.4) | 58 (4.7) |  | | 162 (67.8) | 21 (2.6) | |  | | 297 (66.7) | 87 (6) |
| Normal-like |  | 30 (4.2) | 36 (2.9) |  | | 14 (5.9) | 23 (2.9) | |  | | 26 (5.8) | 256 (17.5) |
| Missing |  | 6 (0.8) | 8 (0.7) |  | |  |  | |  | |  |  |

*Due to larger numbers of METABRIC samples missing stage information, missing samples were not included in calculation of percentages. Samples missing ER status data were excluded from this table across all three studies. ER: estrogen receptor; PR: progesterone receptor; HER2: human epidermal growth factor receptor 2; CBCS: Carolina Breast Cancer Study; TCGA: the Cancer Genome Atlas; METABRIC: Molecular Taxonomy of Breast Cancer International Consortium; IHC: immunohistochemistry; NA: Not available; HER2-E: HER2-enriched.
