# Supplementary material for: Incorporating RNA-based Risk Scores for Genomic Instability to Predict Breast Cancer Recurrence and Immunogenicity in a Diverse Population
Source: Cancer Res Commun. 2023 Jan 5;3(1):12–20. doi: 10.1158/2767-9764.CRC-22-0267 (PMC10035450; doi:10.1158/2767-9764.CRC-22-0267)
Supplement: Supplemental Table ST3 — Supplemental table 3 shows five-year recurrence hazard ratios for immune classes among ER-negative and ER-positive cases in CBCS. [file crc-22-0267-s06.docx]

| **Supplemental Table 3. Five-year recurrence for global immune phenotypes by estrogen receptor status in CBCS Phase 3^a^** | | | | | | | | |
| --- | --- | --- | --- | --- | --- | --- | --- | --- |
|  |  | **ER-Negative Cases (N=385)** | | |  | **ER-Positive Cases (N=859)** | | |
| Immune Phenotype |  | No. of Events/N | Crude HR (95% CI) | HR ^a^  (95% CI) |  | No. of Events/N | Crude HR (95% CI) | HR ^a^  (95% CI) |
| Adaptive-Enriched |  | 30/191 | 1.00 | 1.00 |  | 15/212 | 1.00 | 1.00 |
| Innate-Enriched |  | 37/136 | 1.95  (1.20, 3.17) | 1.78  (1.09, 2.91) |  | 29/307 | 1.40  (0.75, 2.62) | 1.69  (0.90, 3.17) |
| Immune-Quiet |  | 13/58 | 1.59  (0.83, 3.10) | 1.80  (0.92, 3.50) |  | 21/340 | 0.84  (0.43, 1.65) | 1.16  (0.59, 2.30) |

^a^ Results adapted from Hamilton et al., 2022.

CBCS: Carolina Breast Cancer Study; ER: estrogen receptor; HR: Hazard Ratio; 95% CI: 95% Confidence Interval
